# NDE1 positively regulates oligodendrocyte morphological differentiation

**DOI:** 10.1038/s41598-018-25898-4

**Published:** 2018-05-16

**Authors:** Shoko Shimizu, Yugo Ishino, Masaya Tohyama, Shingo Miyata

**Affiliations:** 1 Division of Molecular Brain Science, Research Institute of Traditional Asian Medicine, Kindai University, Osaka-sayama, Osaka, 589-8511 Japan; 2 Osaka Prefectural Hospital Organization, Osaka, 541-8567 Japan

## Abstract

Oligodendrocytes, the myelin-forming cells in the central nervous system (CNS), undergo morphological differentiation characterized by elaborated branched processes to enwrap neuronal axons. However, the basic molecular mechanisms underlying oligodendrocyte morphogenesis remain unknown. Herein, we describe the essential roles of Nuclear Distribution E Homolog 1 (NDE1), a dynein cofactor, in oligodendrocyte morphological differentiation. In the mouse corpus callosum, *Nde1* mRNA expression was detected in oligodendrocyte lineage cells at the postnatal stage. *In vitro* analysis revealed that downregulation of NDE1 by siRNA impaired the outgrowth and extensive branching of oligodendrocyte processes and led to a decrease in the expression of myelin-related markers, namely, CNPase and MBP. In myelinating co-cultures with dorsal root ganglion (DRG) neurons, NDE1-knockdown oligodendrocyte precursor cells (OPCs) failed to develop into MBP-positive oligodendrocytes with multiple processes contacting DRG axons. Immunoprecipitation studies showed that NDE1 interacts with the dynein intermediate chain (DIC) in oligodendrocytes, and an overexpressed DIC-binding region of NDE1 exerted effects on oligodendrocyte morphogenesis that were similar to those following NDE1 knockdown. Furthermore, NDE1-knockdown-impaired oligodendrocyte process formation was rescued by siRNA-resistant wild-type NDE1 but not by DIC-binding region-deficient NDE1 overexpression. These results suggest that NDE1 plays a crucial role in oligodendrocyte morphological differentiation via interaction with dynein.

## Introduction

Oligodendrocytes produce and maintain the myelin sheath that insulates and supports neuronal axons. Axon myelination is essential for the efficient and speedy conduction of action potentials in the CNS^1^.

Developmentally, oligodendrocytes arise from OPCs, which are generated from the ventricular zones of the brain and spinal cord during embryonic stages and after birth^2,3^. Subsequently, OPCs migrate a long distance throughout the CNS and then transform from pre-oligodendrocytes to immature oligodendrocytes^4,5^. They extend multiple membrane processes that enwrap neuronal axons to form myelin sheaths and differentiate into mature myelinating oligodendrocytes^4,5^. These processes require dynamic organization of the cytoskeleton and active transport of myelin components. The oligodendrocyte processes are filled with the microtubule cytoskeleton, which serves as a track for the intracellular transport of organelles, mRNAs, and proteins to specific subcellular compartments^6–8^. Myelin basic protein (*Mbp*) mRNA is transported as RNA granules along microtubules to the peripheral oligodendrocyte processes and then translated locally in the developing myelin sheaths^9–11^. As in neurons, the microtubule motor proteins dynein and kinesin could contribute to oligodendrocyte morphogenesis by targeting and localizing cargo in oligodendrocytes. A mutation of zebrafish *kif1b*, which encodes kinesin, impairs *mbp* mRNA transport and causes ectopic localization of myelin proteins, leading to myelination defects^12^. A mutation of zebrafish *dync1h1*, which encodes the heavy chain subunit of cytoplasm dynein, also causes myelination defects^13^.

We have previously reported that disrupted-in-schizophrenia 1 (DISC1) and DISC1-binding zinc-finger protein (DBZ) are expressed postnatally in oligodendrocyte lineage cells in the corpus callosum, where they regulate oligodendrocyte differentiation^14,15^. *DISC1* is a gene disrupted by a (1;11) (q42.1;q14.3) translocation that has been found to segregate with major psychiatric disorders including schizophrenia (SZ), bipolar disorder (BP), and major depression (MDD) in a Scottish family^16–18^.

In this study, to gain more insight into the molecular mechanisms underlying oligodendrocyte differentiation and myelination, we focused on one of the dynein cofactors, Nuclear Distribution E Homolog 1 (NDE1, also known as NudE). NDE1 is a coiled-coil protein that binds to DISC1 and DBZ^19–21^. It is required for a wide range of cellular processes in neuronal development by modulating dynein function in cooperation with LIS1, another key dynein regulator. These processes include proliferation^22^, neuronal migration^23^, dendrite arborization^24^, and trafficking and localization of cargoes, such as organelles^25^. Previously, Fancy *et al*. have shown that microarray analysis reveals *Nde1* downregulation in the spinal cord of myelination-deficient (Olig2-Cre) × (Floxed dominant-active β-catenin) mice, in which expression of *Mbp*, *Plp*, *Cnp*, and *Mag* are also reduced^26^. These studies raise the possibility that NDE1 plays an important role in oligodendrocyte development. However, NDE1 expression in oligodendrocyte lineage cells and its potential role in oligodendrocyte development remain unknown.

We addressed these issues and report for the first time that NDE1 is expressed in oligodendrocyte lineage cells in the mouse corpus callosum at the postnatal stage and that NDE1 is involved in oligodendrocyte morphological differentiation via interaction with dynein.

## Results

### *Nde1* is expressed in oligodendrocyte lineage cells in the corpus callosum at the postnatal stage

**Figure 1:**
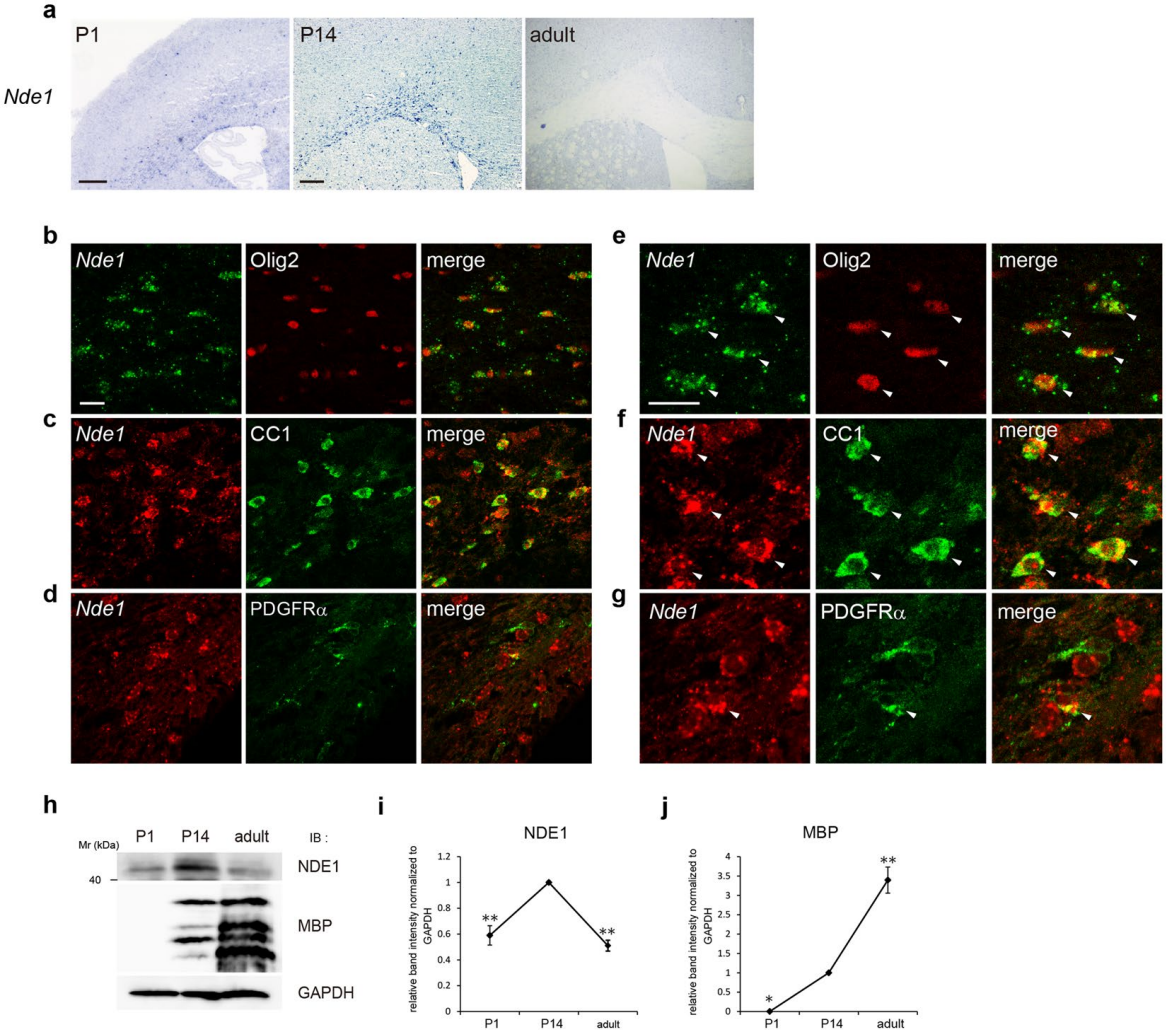
*Nde1* is expressed in oligodendrocyte lineage cells in the mouse carpus callosum. (**a**) *In situ* hybridization analyses of brain sections from P1 (left panel), P14 (middle panel) and adult mice (right panel) with antisense RNA probes to *Nde1* mRNA. (**b**–**g**) Double *in situ* hybridization–immunohistochemistry analyses of brain sections from P14 mice with antisense RNA probes for *Nde1* mRNA and antibodies against Olig2 (**b**,**e**), CC1 (**c**,**f**), and PDGFRα (**d**,**g**) at low magnification (**b**–**d**) and high magnification (**e**–**g**). Arrowheads indicate colocalization. (**h**) Western blot analysis of NDE1 and MBP protein expression in the corpus callosum at different stages. (**i**,**j**) Densitometric quantification of three independent western blot analyses. *P < 0.05; **P < 0.01 versus P14 by Tukey-Kramer post-test following one-way ANOVA (n = 3). Scale bars: (**a**) P1: 0.2 mm, P14 adult: 0.2 mm, (**b**–**g**) 20 µm. Full-length blots are shown in Supplementary Fig. S4a.

Previous studies have reported that *Nde1* is expressed predominantly in proliferating neuronal progenitors and migrating neurons during mouse embryogenesis^22,23^. Despite its functional significance in neuronal development, NDE1 expression and its potential role in oligodendrocyte lineage cells remain poorly understood. To examine if NDE1 is expressed in oligodendrocyte lineage cells, we performed *in situ* hybridization (ISH) analysis of mouse brains using digoxigenin (DIG)-labeled probes for *Nde1*. During development, myelination in the corpus callosum of mice begins at the first postnatal week and peaks postnatally 3–4 weeks after birth^27^. Therefore, we analyzed the expression of *Nde1* mRNA in mouse brains during postnatal development and compared it with adult mice. At postnatal day 1 (P1), ISH analysis revealed that *Nde1*-positive cells were detected in the cortical ventricular zone and corpus callosum (Fig. 1a). At P14, when myelin formation is active, *Nde1*-positive cells were enriched in the corpus callosum, and they were also detected in the subventricular zone (Fig. 1a). On the other hand, cells expressing *Nde1* mRNA in the corpus callosum was hardly detected in adult mice (Fig. 1a).

To confirm whether the cells expressing *Nde1* mRNA in the corpus callosum at P14 were oligodendrocyte lineage cells, we performed ISH combined with immunohistochemical analysis using the antibody for Olig2, an oligodendrocyte lineage cell marker. Most Olig2-immunopositive cells in the corpus callosum expressed *Nde1* mRNA (Fig. 1b,e). To further define the identity of *Nde1* mRNA-expressing cells in the oligodendrocyte developmental stage, *Nde1* mRNA-positive cells were co-labeled with the marker for OPCs or oligodendrocytes, PDGFRα or CC1, respectively. At P14, most *Nde1* mRNA-positive cells expressed CC1 (Fig. 1c,f), whereas a subset of them expressed PDGFRα (Fig. 1d,g).

To examine NDE1 protein expression in the developmental stage, we performed western blot analyses of the corpus callosum at P1 and P14 and of adult mice using antibodies for NDE1 and MBP. Similar to mRNA expression, NDE1 protein expression was detected at P1 and peaked at P14, but it was downregulated in adults (Fig. 1h,i). In contrast, MBP protein expression was hardly detected at P1 and thereafter increased until adulthood (Fig. 1h,j).

These results indicate that *Nde1* is expressed in oligodendrocyte lineage cells in the corpus callosum at the postnatal stage when oligodendrocyte differentiation and myelination are active, suggesting a regulatory role for NDE1 in oligodendrocyte differentiation and/or myelin formation.

### NDE1 knockdown impairs oligodendrocyte process formation ***in vitro***

**Figure 2:**
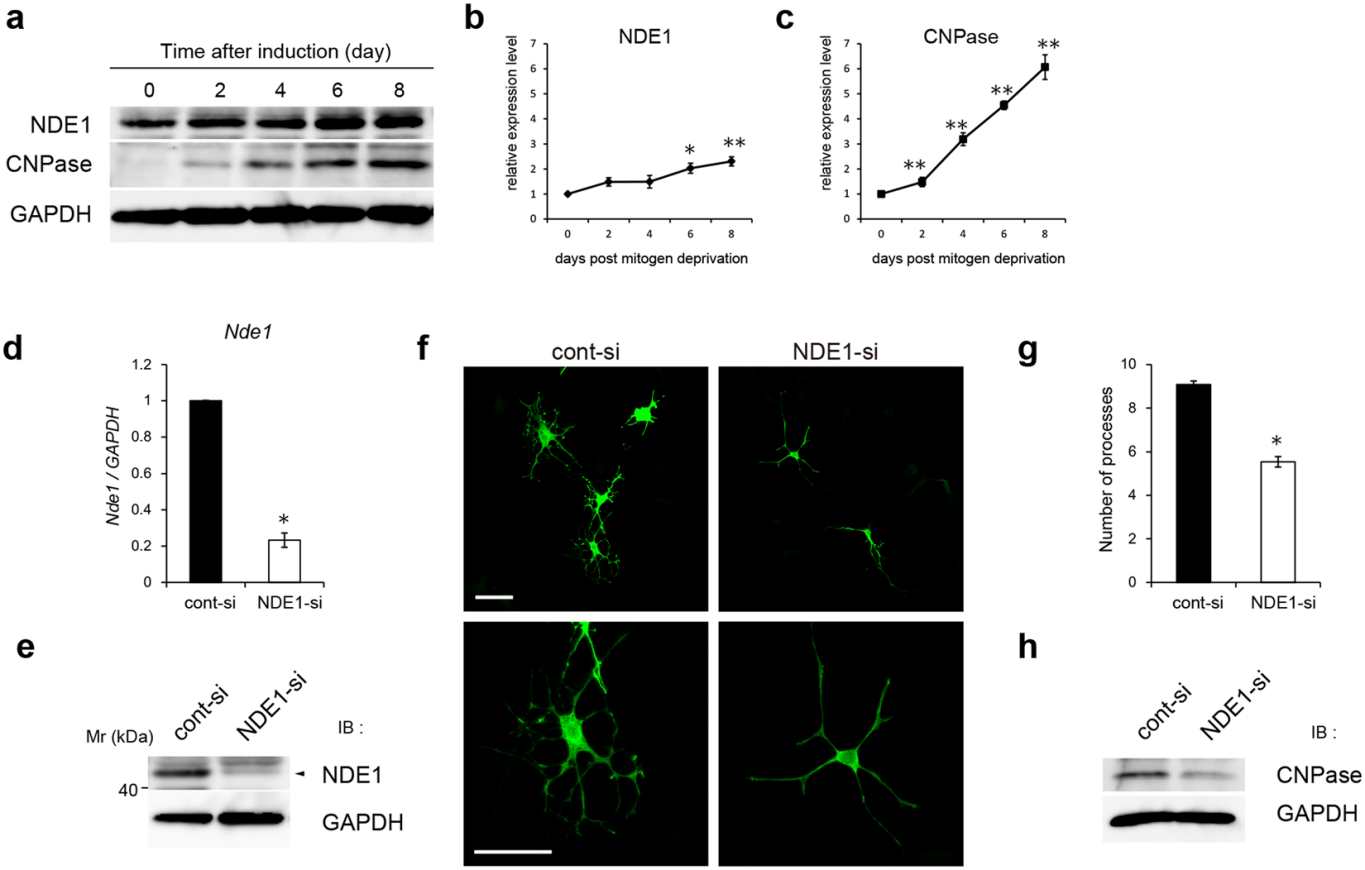
NDE1 is necessary for the morphological differentiation of FBD-102b cells. (**a**) FBD-102b cells were differentiated by reducing serum level. Cell lysates at 0, 2, 4, 6, or 8 days after induction of differentiation were prepared and subjected to western blot analysis using antibodies against NDE1, CNPase, and GAPDH. (**b**,**c**) Quantitative evaluation of NDE1 (**b**) and CNPase (**c**) protein expression level from three independent western blot analyses. *P < 0.05; **P < 0.01 versus day 0 by Tukey-Kramer post-test following one-way ANOVA (n = 3). (**d**) Total RNA was prepared 48 h after siRNA transfection. Expression of *Nde1* was quantified using qRT-PCR. *P < 0.01 versus cont-si (n = 3). (**e**) Lysates were prepared from cells harvested after 48 h after siRNA transfection and expression of NDE1 was assessed by western blotting. (**f**) FBD-102b cells were cotransfected with NDE1 siRNA and GFP-plasmid (NDE1-si) or with control siRNA and GFP-plasmid (cont-si). At 48 h after induction of differentiation, cells were immunostained with antibodies against GFP. (**g**) The number of processes of the cells transfected with siRNA and GFP is shown. *P < 0.05 versus cont-si (n = 3). (**h**) Lysates were prepared from cells harvested at 96 h after induction of differentiation and expression of CNPase was assessed by western blotting. Scale bar: (**f**) 50 µm. Full-length blots are shown in Supplementary Fig. S4b–d.

We explored the regulatory role of NDE1 in oligodendrocyte differentiation *in vitro* assays using FBD-102b cells derived from fetal brains of P53-deficient mice as OPCs model^28^. It has been reported that FBD-102b cells are induced to differentiate into oligodendrocytes with highly branched multipolar processes by reducing serum levels in the culture media^28^. Thus, we examined the expression of NDE1 and the oligodendrocyte marker CNPase protein during the *in vitro* FBD-102b cell morphological differentiation process. CNPase protein expression was increased time dependently after induction of differentiation, indicating the progression of differentiation (Fig. 2a,c). NDE1 protein expression gradually increased in a time-dependent fashion after the induction of differentiation (Fig. 2a,b). These results indicate that NDE1 expression is abundant in the late stage of oligodendrocyte differentiation.

A previous study indicated that the loss of *Drosophila* NudE function results in abnormal dendrite arborization^24^. To evaluate the role of endogenous NDE1 in the morphological differentiation of FBD-102b cells, cells were transfected with siRNA targeting *Nde1* mRNA (NDE1-si) or control siRNA (cont-si) and differentiated. At 48 h after transfection, *Nde1* mRNA expression was significantly reduced in NDE1-si–transfected cells (Fig. 2d). Furthermore, NDE1 protein expression was significantly decreased in NDE1-si–transfected cells (Fig. 2e). These results confirm the effective targeting of *Nde1* mRNA by siRNA. To quantitatively evaluate the effect of NDE1 downregulation on cell morphology, cells were co-transfected with siRNA and GFP expression vectors. At 48 h after induction of differentiation, immunostaining analysis revealed that cont-si–treated cells displayed differentiated morphology with multiple primary and extensive secondary processes (Fig. 2f), whereas NDE1-si–treated cells showed an immature morphology with simple processes (Fig. 2f). Knockdown of NDE1 reduced the number of processes in cells transfected with NDE1-si (5.5 ± 0.24) compared with those transfected with cont-si (9.1 ± 0.16) (Fig. 2g). Western blot analysis revealed that NDE1 knockdown resulted in a decrease in the protein expression of CNPase, which is required for process formation by binding to tubulin heterodimers and driving microtubule assembly^29^ (Fig. 2h). These results suggest that NDE1 is required for process formation during the differentiation process of FBD-102b cells.

We next examined the effect of endogenous NDE1 knockdown on the complexity of the processes using primary cultured oligodendrocytes. OPCs were isolated from the postnatal rat cortex and induced to differentiate by withdrawing PDGF from the culture media^15^. During oligodendrocyte differentiation, bipolar OPCs undergo dynamic morphological changes to pre-myelinating oligodendrocytes with highly branched processes.

**Figure 3:**
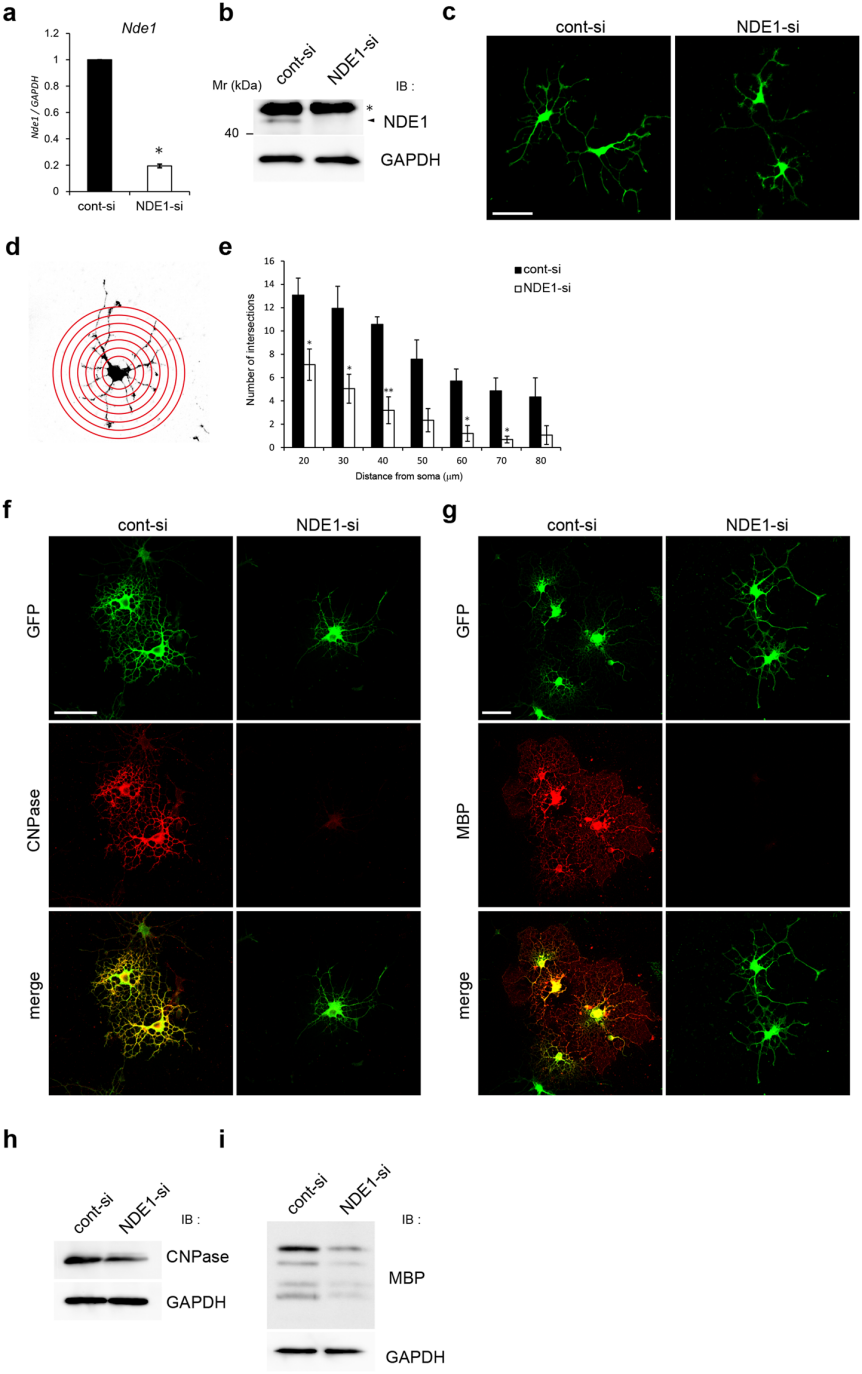
Downregulation of NDE1 impairs process formation in primary cultured oligodendrocytes. (**a**) Total RNA was prepared 48 h after transfection. Expression of *Nde1* was quantified using qRT-PCR. *P < 0.01 versus cont-si (n = 3). (**b**) Lysates were prepared from cells harvested 48 h after transfection and expression of NDE1 was assessed by western blotting. Asterisk indicates non-specific band. (**c**) Primary cultured OPCs were transfected with NDE1 siRNA and GFP-plasmid (NDE1-si) or with control siRNA and GFP-plasmid (cont-si). At 48 h after induction of differentiation, cells were immunostained with antibodies against GFP. (**d**,**e**) Process complexity and branching were evaluated by Sholl analysis. A series of concentric circles was superimposed around the center of the cell and the average number of intersections at each radius was determined. The following parameters were used: starting radius 20 µm, ending radius 80 µm, and step size 10 µm (**d**). The number of intersections made by oligodendrocytes differentiated for 48 h at each radius is shown. Results are the mean of three independent experiments. More than 50 images were obtained from three independent experiments. *P < 0.05; **P < 0.01 versus cont-si by Bonferroni’s post-test following two-way ANOVA (n = 3) (**e**). (**f**,**g**) At 96 h (**f**) and 120 h (**g**) after induction of differentiation, cells were immunostained with antibodies against GFP and CNPase (**f**) or GFP and MBP (**g**). (**h**,**i**) Lysates were prepared from cells harvested 96 h (**h**) and 120 h (**i**) after differentiation and expression of CNPase (**h**) and MBP (**i**) was assessed by western blotting. Scale bars: (**c**,**f**,**g**) 50 µm. Full-length blots are shown in Supplementary Fig. S4e–g.

To examine the effect of NDE1 knockdown on the morphological differentiation of primary cultured oligodendrocytes, OPCs were co-transfected with siRNA and GFP expression vectors. At 48 h after induction of differentiation, *Nde1* mRNA and protein expression was significantly reduced in NDE1-si–treated cells (Fig. 3a,b). Control cells (co-transfected with cont-si and GFP) exhibited an elaborate morphology with highly branched thick primary and thinner secondary processes (Fig. 3c). On the other hand, NDE1 knockdown cells (co-transfected with NDE1-si and GFP) displayed less complex morphology with simple and shorter processes (Fig. 3c). The morphological complexity of oligodendrocyte processes was quantified by Sholl analysis (Fig. 3d). NDE1 knockdown cells developed fewer and shorter branches, producing a less elaborate network of processes (Fig. 3e). At 96–120 h after induction of differentiation, control cells developed membranous sheets and expressed CNPase and MBP protein in the soma and peripheral processes, while NDE1 knockdown cells rarely extended elaborated structures and expressed low levels of these proteins in the soma (Fig. 3f,g). Western blot analysis also confirmed the decrease in CNPase and MBP protein expression by NDE1 knockdown (Fig. 3h,i). These results indicate that knockdown of NDE1 impairs the outgrowth and extensive branching of oligodendrocyte processes and leads to a decrease in the protein expression of CNPase and MBP in primary cultured oligodendrocytes, suggesting that NDE1 is necessary for sequential and morphological differentiation of oligodendrocytes.

### Knockdown of NDE1 impairs oligodendrocyte myelination in co-cultures of oligodendrocytes and DRG neurons

**Figure 4:**
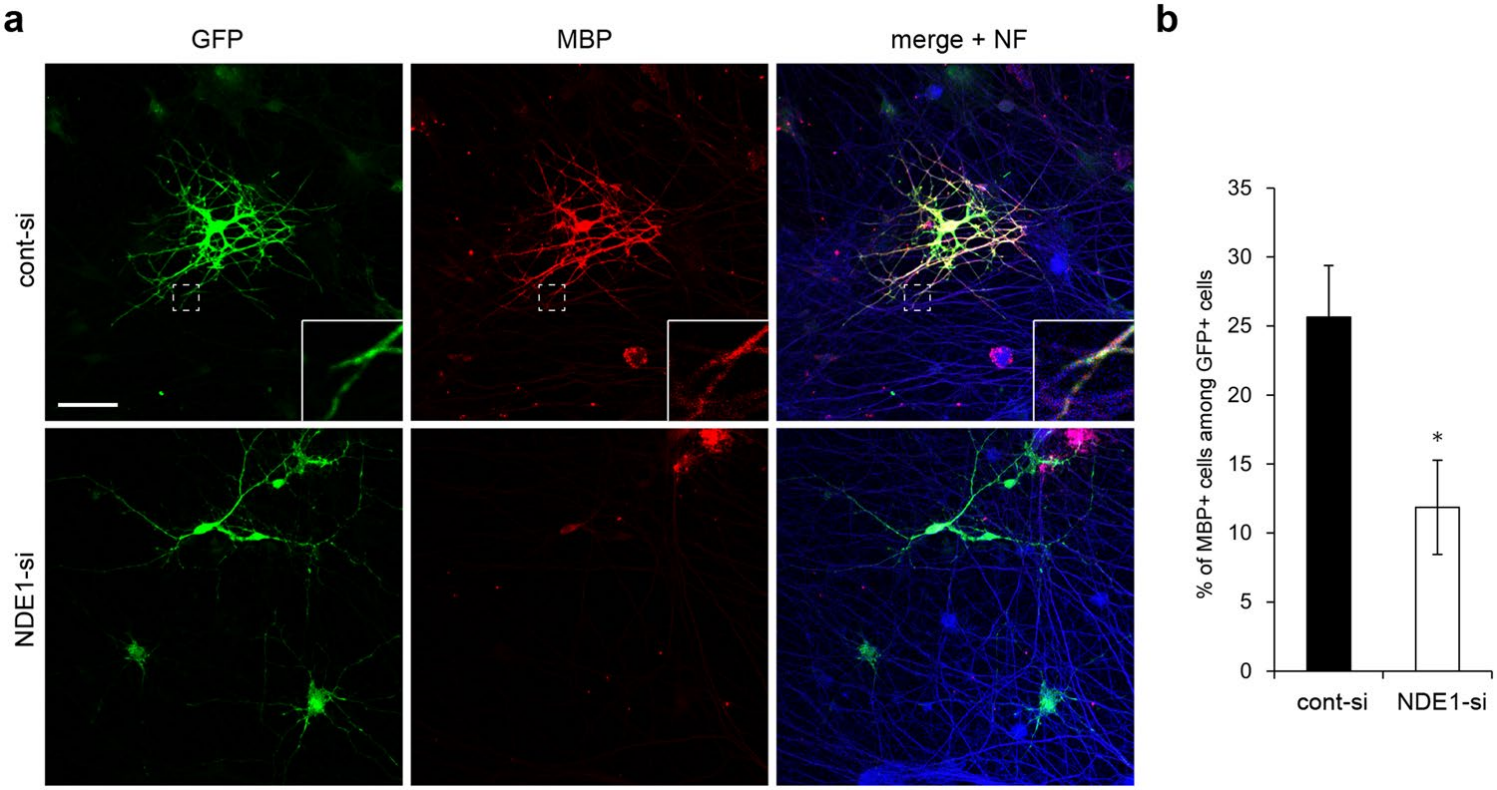
Effect of NDE1 downregulation on neuron–oligodendrocyte contact formation in myelinating co-cultures. OPCs were transfected with NDE1 siRNA and GFP-plasmid (NDE1-si) or with control siRNA and GFP-plasmid (cont-si), added to DRG neurons, and co-cultured for 10 days. (**a**) Immunostaining was performed using antibodies against GFP, MBP and neurofilament protein NF. (**b**) Quantification of MBP+/GFP+ ccells from the data shown in (**a**). Results are the mean of three independent experiments. More than 100 cells were counted in one culture. *P < 0.05 versus cont-si (n = 3). Scale bar: (**a**) 50 µm.

We next examined the effect of NDE1 knockdown on axon–oligodendrocyte interaction and myelination using cocultures of oligodendrocytes and DRG neurons. To monitor cell morphology, OPCs were co-transfected with siRNA and GFP expression vectors by nucleofection. These transfected OPCs were added to DRG neurons for 10 days. The DRG axonal processes were labeled with the high-molecular-weight neurofilament protein NF-H, and oligodendrocytes were co-labeled with GFP and MBP. As shown in Fig. 4a, over 25% of control cells (co-transfected with cont-si and GFP) exhibited multipolar morphology with elaborated processes and expressed high levels of MBP, which indicated that these cells had differentiated into mature oligodendrocytes (Fig. 4a). It was demonstrated that these cells had wrapped their processes around NF-positive DRG axons (Fig. 4a, inset). In contrast, most NDE1 knockdown cells (co-transfected with NDE1-si and GFP) displayed immature morphology with poor and thin processes and expressed low levels of MBP (Fig. 4a). These processes touched and elongated along the DRG axons, but failed to form myelin sheaths (Fig. 4a). As shown in Fig. 4b, the proportion of MBP-positive to GFP-positive cells in NDE1-si treated cultures was lower than in cont-si treated cultures (cont-si: 25.6 ± 3.75% vs. NDE1-si: 11.9 ± 3.41%) (Fig. 4b). Furthermore, these co-cultures showed that the proportion of final differentiated MBP-positive cells after 21 days is significantly lower following NDE1 knockdown (Supplementary Fig. S1a,b).

These experiments suggest that NDE1 downregulation impairs the axon–oligodendrocyte interaction, and leads to abnormalities in the early myelination process of oligodendrocytes in myelinating co-cultures.

### NDE1 interacts with cytoplasmic dynein through its intermediate chain (DIC) in oligodendrocytes

**Figure 5:**
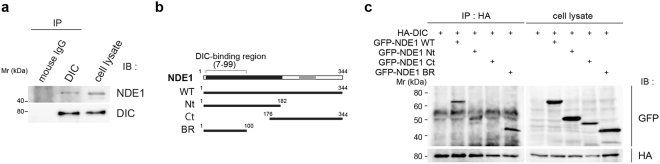
NDE1 interacts with the intermediate chain of cytoplasmic dynein (DIC) in oligodendrocytes. (**a**) Immunoprecipitation assay with DIC antibodies using FBD-102b cell lysate. The precipitated proteins and inputs were subjected to western blot analysis using anti-NDE1 antibodies. (**b**) Schematic representations of the NDE1 deletion fragments used in this study. NDE1 contains the N-terminal coiled-coil domain (black rectangle) and the C-terminal α-helix (gray rectangle). The DIC-binding site of NDE1 from the literature is between amino-acid residues 7 and 99^34^. (**c**) Binding of NDE1 and its deletion fragments to DIC. HEK293T cells were co-transfected with HA-tagged DIC and GFP-tagged full-length wild-type NDE1 (NDE1 WT) or GFP-tagged NDE1 deletion fragments shown in (**b**). Immunoprecipitates by anti-HA antibodies were subjected to western blot analysis using anti-GFP antibodies. Full-length blots are shown in Supplementary Fig. S4h,i.

Previous studies have reported that NDE1 regulates cytoplasmic dynein function in several cellular processes^22–24,30^. We next examined whether NDE1 is involved in process formation and myelination by binding to dynein in oligodendrocytes. Cytoplasmic dynein is a large protein complex that consists of two dynein heavy chains (DHCs), two DICs, four light intermediate chains (DLICs), and several light chains (DLCs)^31,32^. NDE1 interacts with the cytoplasmic dynein predominantly through its DIC^33^. To examine the association of NDE1 with dynein in oligodendrocytes, we performed an immunoprecipitation assay using FBD-102b cells. The cell lysates were prepared and immunoprecipitated with anti-DIC antibody. Then, western blot analysis using an anti-NDE1 antibody revealed the interaction between endogenous NDE1 and DIC in FBD-102b cells (Fig. 5a). These results suggest that NDE1 may play a role in process formation and myelination by binding to dynein through its DIC during oligodendrocyte differentiation.

### Overexpression of DIC-binding region of NDE1 impairs process formation in oligodendrocytes

To evaluate the physiological role of the NDE1–dynein interaction during oligodendrocyte differentiation, we first confirmed the interaction of the DIC-binding domain of NDE1 with DIC in mammalian cells. A previous study using a pull-down assay has revealed that NDE1 interacts with DIC via the N-terminal region of the coiled-coil domain of NDE1 (amino acids 7–99) in *Xenopus* egg extract^34^ (Fig. 5b). Thus, we further constructed GFP-tagged mouse NDE1 expression vectors of the N-terminal region (NDE1 Nt, amino acids 1–182), C-terminal region (NDE1 Ct, amino acids 176–344), and DIC-binding region (NDE1 BR, amino acids 1–100), and performed coimmunoprecipitation analysis using HEK293T cells that were co-transfected with HA-tagged DIC and GFP-tagged full-length wild-type NDE1 (NDE1 WT) or GFP-tagged NDE1 deletion mutants (Fig. 5b). We found that the NDE1 WT, NDE1 Nt, and NDE1 BR retain the ability to co-precipitate with DIC, whereas NDE1 Ct cannot bind DIC (Fig. 5c). In agreement with previous reports, these results suggest that the NDE1 BR region is essential for interaction with DIC. This domain is expected to function as a dominant-negative form of NDE1 through the inhibition of binding between NDE1 and DIC in mammalian cells.

**Figure 6:**
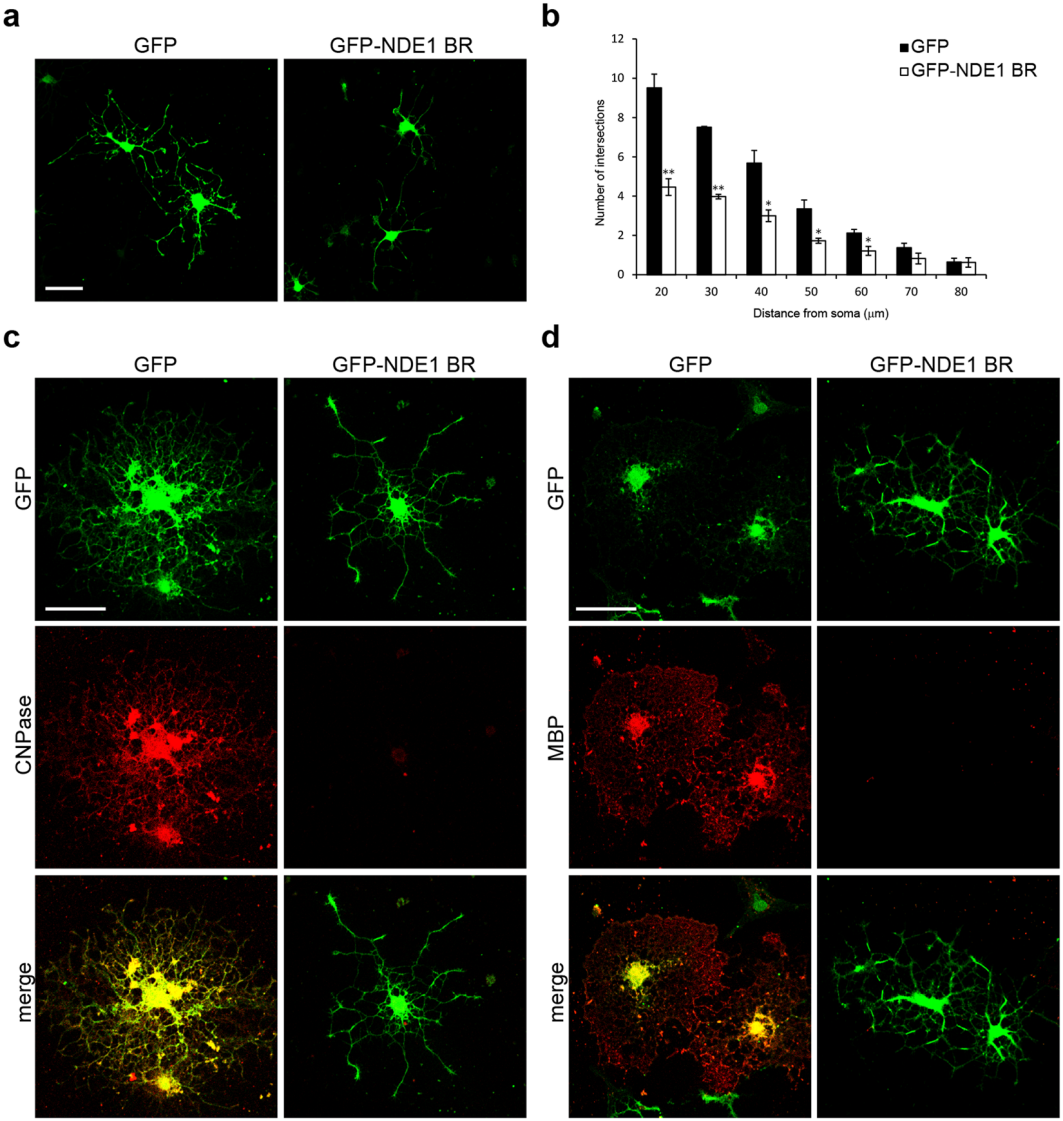
Overexpressed DIC-binding region of NDE1 impairs process formation in primary cultured oligodendrocytes. (**a**) Primary cultured oligodendrocytes were transfected with GFP-NDE1 BR or GFP. At 48 h after induction of differentiation, cells were immunostained with anti-GFP. (**b**) Process complexity and branching are evaluated by Sholl analysis. Results are the mean of three independent experiments. More than 50 images in total were obtained from three independent experiments. *P < 0.05; **P < 0.01 versus GFP by Bonferroni’s post-test following two-way ANOVA (n = 3) (**b**). (**c**,**d**) At 96 h after induction of differentiation, cells were immunostained with antibodies against GFP and CNPase (**c**). At 120 h after induction of differentiation, cells were immunostained with antibodies against GFP and MBP (**d**). Scale bars: (**a**,**c**,**d**) 50 µm.

To examine the effect of the inhibition of the NDE1–dynein interaction on process formation in primary cultured oligodendrocytes, cells were transiently transfected with GFP-NDE1 BR or GFP. At 48 h after differentiation, the cells expressing GFP displayed elaborate morphology with highly branched thick primary and thinner secondary processes (Fig. 6a). On the other hand, the cells expressing GFP-NDE1 BR showed less complex and differentiated morphology with thinner and shorter processes (Fig. 6a). Sholl analysis revealed significant differences in process complexity between cells expressing GFP-NDE1 BR and cells expressing GFP (Fig. 6b). Furthermore, at 96–120 h after differentiation, GFP-expressing cells developed membranous sheets and expressed CNPase and MBP protein in the soma and peripheral processes (Fig. 6c,d), whereas GFP-NDE1 BR–expressing cells rarely extended elaborated structures and hardly expressed CNPase and MBP protein (Fig. 6c,d). We also confirmed that process formation is inhibited by the overexpressed NDE1 BR region in FBD-102b cells (Supplementary Fig. S2a–c).

These results suggest that the interaction of NDE1 and dynein plays a crucial role in process formation during oligodendrocyte differentiation.

### Overexpression of DIC-binding region of NDE1 impairs oligodendrocyte myelination in co-cultures of oligodendrocytes and DRG neurons

**Figure 7:**
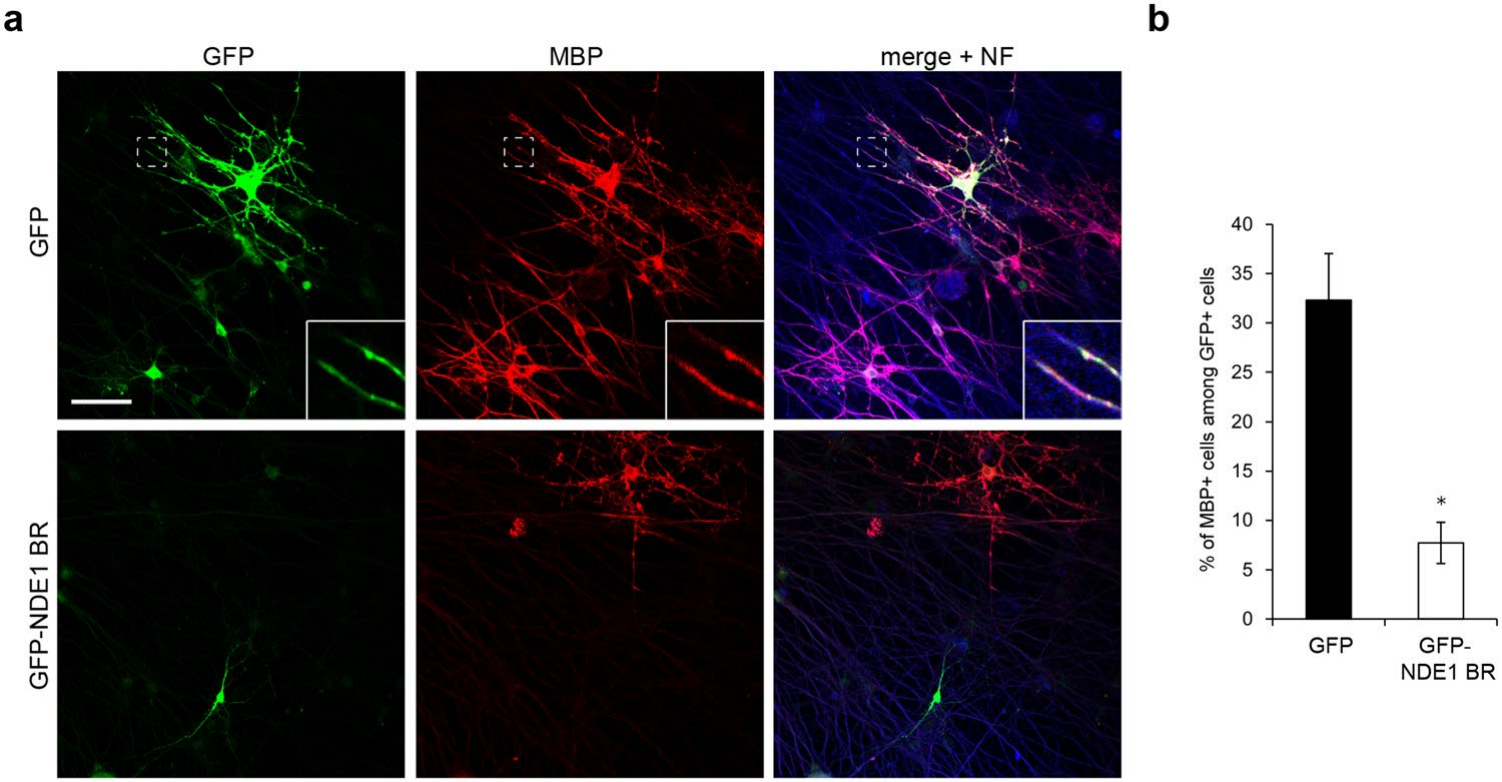
Effect of overexpressed DIC-binding region of NDE1 on neuron–oligodendrocyte contact formation in myelinating co-cultures. OPCs were transfected with GFP-NDE1 BR or GFP, added to DRG neurons, and co-cultured for 10 days. (**a**) Immunostaining was performed using antibodies against GFP, MBP and neurofilament protein NF. (**b**) Quantification of MBP+/GFP+ cells from the data shown in (**a**). Results are the mean of three independent experiments. More than 100 cells were counted in each culture. *P < 0.01 versus GFP (n = 3). Scale bar: (**a**) 50 µm.

To evaluate the physiological role of the NDE1–dynein interaction during the early phase of myelination, we examined the effect of overexpression of NDE1 BR on the axon–oligodendrocyte interaction and myelination using co-cultures of oligodendrocytes and DRG neurons. OPCs were transfected with GFP-NDE1 BR or GFP, and then added to DRG neurons. 10 days after co-culture, more than 30% of GFP-expressing cells contacted DRG axons via their processes (Fig. 7a, inset), and expressed MBP protein throughout the cell soma and peripheral processes that were extending along DRG axons, indicating that these cells had developed into myelin-forming oligodendrocytes (Fig. 7a). On the other hand, most GFP-NDE1 BR–expressing cells displayed undifferentiated morphology with stunted and poorly arborized processes and hardly expressed MBP protein, indicating that these cells failed to myelinate DRG axonal processes (Fig. 7a). As shown in Fig. 7b, the proportion of MBP-positive cells to GFP-positive cells was significantly lower in GFP-NDE1BR–treated cultures (GFP: 32.3 ± 4.74% vs. GFP-NDE1 BR: 7.7 ± 2.10%) (Fig. 7b). These results suggest that the interaction of NDE1 and dynein plays a crucial role in axon–oligodendrocyte contact formation and the early process of myelination in co-cultures of oligodendrocytes and DRG neurons.

### Impaired oligodendrocyte process formation is not rescued by DIC-binding region-deficient NDE1

**Figure 8:**
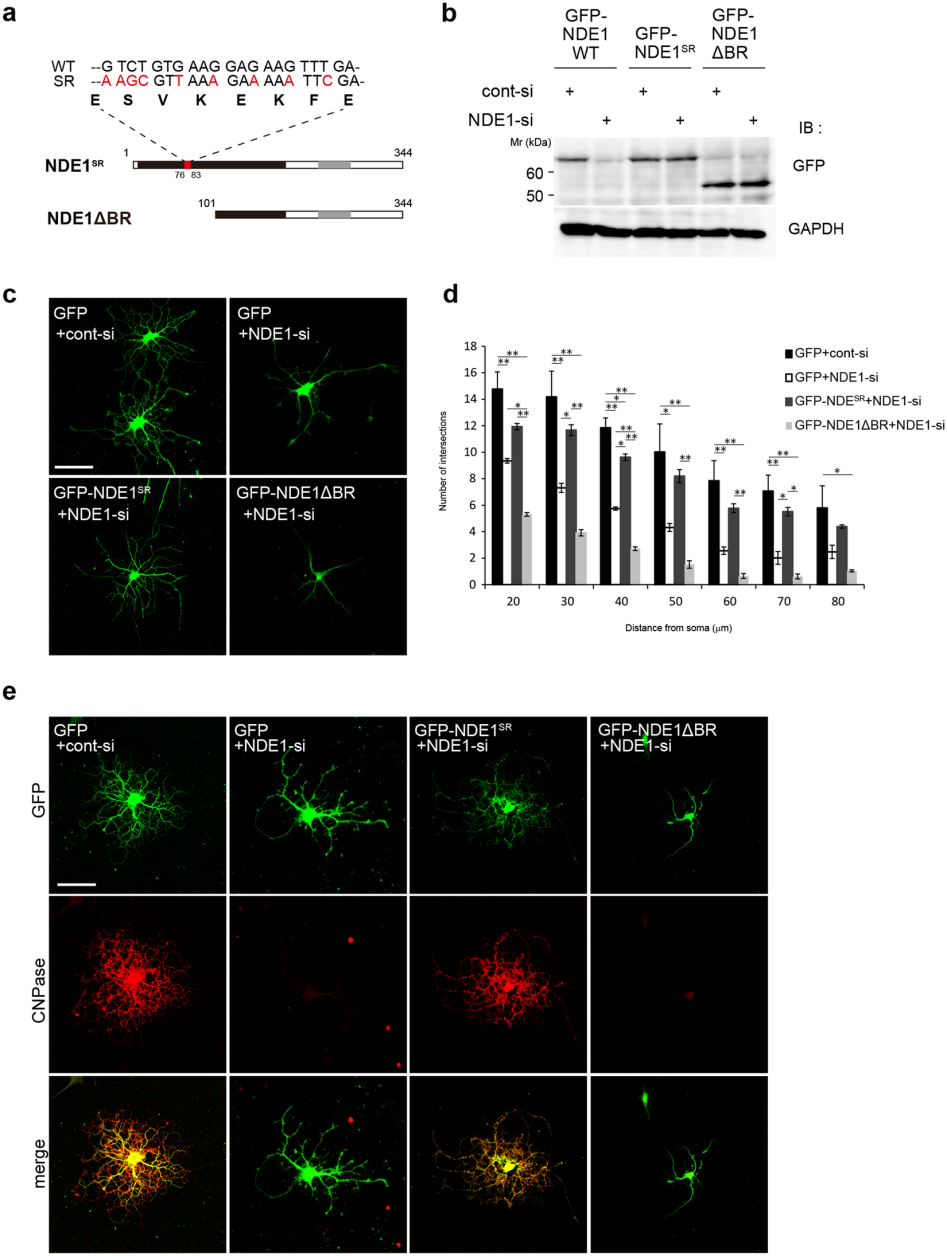
Impaired oligodendrocyte process formation is not rescued with a DIC-binding region-deficient NDE1 in primary cultured oligodendrocytes. (**a**) An illustration of the siRNA-resistant wild-type NDE1 (NDE1^SR^) that has a modified NDE1 siRNA target region (red box) without changing the amino acid sequence, and an NDE1 deletion mutant that lacks the DIC-binding region (NDE1ΔBR). NDE1ΔBR does not contain the NDE1 siRNA target sequence. (**b**) HEK293T cells were co-transfected with siRNA and GFP-NDE1 WT, GFP-NDE1^SR^, or GFP-NDE1ΔBR. At 48 h after post-transfection, cell lysates were prepared and expression of GFP was assessed by western blotting. (**c**) OPCs were co-transfected with NDE1 siRNA or control siRNA and GFP, GFP-NDE1^SR^, or GFP-NDE1ΔBR in combination. At 48 h after induction of differentiation, cells were immunostained with antibodies against GFP. (**d**) Process complexity and branching are evaluated by Sholl analysis. Results are the mean of three independent experiments. More than 50 images were obtained from three independent experiments. *P < 0.05; **P < 0.01 by Bonferroni’s post-test following two-way ANOVA (n = 3). (**e**) At 96 h after differentiation, cells were immunostained with antibodies against GFP and CNPase. Scale bars: (**c**,**e**) 50 µm. Full-length blots are shown in Supplementary Fig. S4j.

Based on our *in vitro* findings, NDE1 appeared to be involved in oligodendrocyte morphological differentiation via interaction with dynein. To confirm these results, we performed a rescue experiment in NDE1 knockdown cells. We generated GFP-tagged expression vectors of siRNA-resistant wild-type NDE1 that has a modified NDE1 siRNA target sequence without changing the amino acid sequence (NDE1^SR^) and an NDE1 deletion mutant that lacks the DIC-binding region (NDE1ΔBR) (Fig. 8a). First, we checked the expression levels of overexpressed NDE1 in HEK293T cells co-transfected with siRNA and GFP-tagged NDE1 WT, NDE1^SR^, or NDE1ΔBR. Western blot analysis showed that GFP-NDE1 WT was significantly knocked down by NDE1-si, but GFP-NDE1^SR^ and GFP-NDE1ΔBR were not (Fig. 8b).

In FBD-102b cells, knockdown of NDE1 led to decreased process numbers, and this phenotype was rescued by expression of GFP-NDE1^SR^ but not GFP-NDE1ΔBR (Supplementary Fig. S3a,b). Furthermore, in primary cultured oligodendrocytes, NDE1 knockdown led to a significant decrease in complexity, and these loss of function phenotypes were rescued by expression of GFP-NDE1^SR^, but not GFP- NDE1ΔBR (Fig. 8c,d). At 96 h after differentiation, CNPase protein expression was decreased by NDE1 knockdown, and the expression was recovered by GFP-NDE1^SR^ but not GFP-NDE1ΔBR co-transfection (Fig. 8e). These results provide further evidence that NDE1 is involved in oligodendrocyte morphological differentiation via interaction with dynein.

## Discussion

The major findings of this study were as follows: (1) *Nde1* mRNA is expressed in oligodendrocyte lineage cells in the corpus callosum at the postnatal stage; (2) NDE1 downregulation impairs process formation in oligodendrocytes, and results in the failure of neuron–oligodendrocyte contact formation and myelination; (3) NDE1 interacts with the DIC of cytoplasmic dynein in oligodendrocytes; (4) overexpression of a DIC-binding region of NDE1 (NDE1 BR) in oligodendrocytes inhibits process formation and myelination; and (5) impaired oligodendrocyte process formation is rescued with siRNA-resistant wild-type NDE1 (NDE1^SR^), but not with DIC-binding region-deficient NDE1 (NDE1ΔBR) overexpression in NDE1-downregulated oligodendrocytes. Taken together, our data indicate that NDE1 plays a crucial role in process formation and myelination by binding to dynein in oligodendrocytes.

Previous studies have reported that *Nde1* is primarily expressed in the neural progenitors of the cortical ventricular zone and newly formed cortical plate neurons during embryogenesis and is dramatically downregulated in the perinatal period when the cortical neurogenesis is completed^22,23^. More recently, analysis of data from the Human Brain Transcriptome project revealed that *Nde1* expression is dramatically decreased at birth but is slightly upregulated in some brain regions after birth^35^. In this study, we first show that *Nde1* mRNA is expressed in oligodendrocyte lineage cells in the corpus callosum of mice at the early postnatal stage when oligodendrocyte differentiation and myelin formation are active, and that the expression is downregulated at adulthood. These results suggest that NDE1 has a developmental role in oligodendrocyte lineage cells.

Our *in vitro* analysis reveals that NDE1 downregulation by siRNA impaired the outgrowth and extensive branching of oligodendrocyte processes. NDE1 is a known dynein cofactor that regulates dynein activity by recruiting LIS1 to dynein. Together, they form a triple complex, inducing a force-state and promoting the movement of dynein along the microtubules^36^. Dynein is essential for proper morphogenesis, acting on well-characterized minus-end-directed transport of cargoes such as mRNA, proteins, and organelles from distal tips of processes to the cell soma. Dynein is also involved in the modulation of microtubule dynamics at the cell cortex by stabilization of microtubule plus ends and tethering them to the cell periphery^37^. Dynein could function similarly in oligodendrocytes and contribute to the proper oligodendrocyte morphogenesis. In cultured mouse oligodendrocytes, cytoplasmic dynein is localized in the cell body and along microtubules in the peripheral processes^38^. A recent study has reported that mutation of zebrafish cytoplasm dynein causes a defect in myelination^13^. These studies suggest the possibility that dynein plays a pivotal role in oligodendrocyte morphogenesis. Combined with these studies, our data indicate that impaired process formation by NDE1 downregulation in oligodendrocytes could be attributed to dysregulation of dynein motor functions. A previous report has shown that Drosophila NudE plays an important role in neuronal morphogenesis by regulating dynein activity^24^, suggesting the possibility that NDE1 has common functional roles between neurons and oligodendrocyte lineage cells. This study has shown that Drosophila NudE associates with Golgi outposts, which shape dendrite morphology by functioning as sites of acentrosomal microtubule nucleation in neurons, and that loss of the Drosophila NudE gene affects the transport of Golgi outposts, resulting in abnormal dendrite arborization^24^. In mammals, NDE1 is reported to be involved in dynein-dependent Golgi organization and localization^30^. Interestingly, a previous study has reported that the Golgi complex resides not only in the perikaryon but also in the processes in primary cultures of rat oligodendrocytes^39^. As in neurons, the dynein–NDE1 complex in oligodendrocytes could function in the targeting and localization of the Golgi complex in peripheral processes. However, it remains unknown whether Golgi outposts could be present in branching points of peripheral processes in the oligodendrocytes, and represent a source for acentrosomal microtubule nucleation. Additional information about the molecular mechanisms underlying oligodendrocyte morphological differentiation must be obtained.

Consistent with previous studies, our data show that the N-terminal coiled-coil domain of NDE1, residues 1–100, is sufficient to interact with DIC^34^. Furthermore, our data reveal that NDE1 interacts with DIC in the oligodendrocyte lineage cells, and overexpression of NDE1 BR inhibits oligodendrocyte morphological differentiation, potentially acting as a dominant negative form of NDE1 through inhibition of the binding between full-length NDE1 and DIC. However, NDE1 is reported to interact with the coiled-coil N-terminus of DIC and compete with dynactin, which is an essential cofactor of the cytoplasmic dynein, for a common binding region of DIC^33^. A recent study has reported that mutation of zebrafish *actr10*, which encodes the Arp11 subunit of dynactin, causes hypomyelination^40^. Although it remains unclear whether NDE1 could compete with dynactin for binding to DIC in oligodendrocyte lineage cells, we could not exclude the possibility that impaired oligodendrocyte morphogenesis by the NDE1 BR expression could be partially attributed to the inhibition of dynactin-DIC interaction. Additionally, our study demonstrates that impaired oligodendrocyte morphological differentiation by siRNA was rescued by NDE1^SR^ but not by NDE1ΔBR overexpression. These results support the conclusion that the interaction between the N-terminal site on NDE1 and DIC is essential for oligodendrocyte morphological differentiation. NDE1 is reported to function as a molecular tether that facilitates the interaction between dynein and LIS1 in dendrite morphogenesis^24^. To further determine the role of NDE1-dynein on oligodendrocyte development, it is important to examine the expression and function of other dynein cofactors in oligodendrocytes.

Copy number variants at the chromosome 16p13.11 locus, which contains the *Nde1* gene, have been implicated in a range of neurodevelopmental disorders, including autism, attention deficit hyperactivity disorder, and SZ^41–45^. Genetic association studies of NDE1 with SZ using single-nucleotide polymorphisms and haplotypes have been reported^46^. Recently, multiple lines of evidence have implicated oligodendrocytes and myelin abnormalities in neuropsychiatric diseases including SZ, BP, and MDD^47,48^. In the future, establishing oligodendrocyte lineage cell-specific NDE1 knockout mice will be essential to explore the role of NDE1 in oligodendrocyte differentiation and myelination, which might contribute to the elucidation of molecular mechanisms underlying the pathology of the above-mentioned psychiatric disorders.

## Methods

### Animals

C57BL/6 mice of either sex were used for the construction of cDNA, primary culture of purified DRG neurons, and ISH. Wistar rats of either sex were used for *in vitro* oligodendrocyte primary culture. All animal experiments were performed in accordance with protocols approved by the Animal Ethics Committee of Kindai University.

### Chemicals and antibodies

The primary antibodies used in this study were as follows: anti-NDE1 (Proteintech, 1:500); anti-Olig2 (Millipore, 1:100); anti-PDGFRα (Santa Cruz, 1:100); anti-CC1 (Millipore, 1:100); anti-CNPase (Sigma, 1:300); anti-MBP (Millipore, 1:300); anti-Neurofilament 200 (NF, 1:300) (Sigma); anti-GFP (Santa Cruz, 1:300); anti-copGFP (Evrogen, 1:500); anti-HA (Sigma, 1:500); anti-dynein, 74-kDa intermediate chains, cytoplasmic (Millipore, 1:500); and anti-GAPDH (Santa Cruz, 1:300). The secondary antibodies were as follows: horseradish peroxidase (HRP)-conjugated anti-immunoglobulin G (IgG) (Cell Signaling, 1:1000); and Alexa Fluor 488-conjugated anti-IgG, Alexa Fluor 568-conjugated anti-IgG, and Alexa Fluor 350-conjugated anti-IgG (Molecular Probes, 1:1000).

### Small interfering RNA design

We obtained siRNAs from Sigma. The siRNA sequence for knockdown of NDE1 (NDE1-si) was: 5′-GTCTGTGAAGGAGAAGTTTGA-3′ (mouse and rat). A scrambled sequence was used as a control (cont-si): 5′-GGCGCGCTTTGTAGGATTCGA-3′.

### Plasmids

Murine NDE1 cDNA fragments were amplified by PCR using the forward/reverse primers 5′- ATGGAGGACTCGGGAAAGACCTTTG-3′/5′-GGGCGCAGTTCTCATTGGTCCTAAA-3, and subcloned into the pGEM-T vector (Promega). For the construction of GFP-tagged NDE1 expression vectors, full-length wild-type mouse NDE1 protein (NDE1 WT: amino acids 1–344), N-terminal region (NDE1 Nt: amino acids 1–182), C-terminal region (NDE1 Ct: amino acids 176–344), and DIC-binding region (NDE1 BR: amino acids 1–100) were amplified from pGEM-T/NDE1 vector using following forward/reverse primers: NDE1 WT, 5′-CTGCTCGAGATGGA GGACTCGGGAAAGAC-3′/5′-CAGGAATTCCTAAAGCAAAAGCTTGACCA-3′, NDE1 Nt, 5′-CTGCTCGAGATGGAGGACTCGGGAAAGAC-3′/5′-CAGGAATTCCTACACAGCCAATTCCTGCC-3′, NDE1 Ct, 5′-CTGCTCGAGCTGCGGCAGGAATTGGCTGT-3′/5′- CAGGAATTCCTAAAGCAAAAGCTTGACCA-3′, and NDE1 BR, 5′-CTGCTCGAGATGGAGGACTCGGGAAAGAC-3′/5′-CAGGAATTCCTACAGGTCATCCTCCAAGG-3′. The PCR fragments were digested with XhoI and EcoRI and resulting DNA fragments were inserted into XhoI/EcoRI site of pEGFP-C3 (Clontech) vectors. Expression vectors of NDE1 siRNA-resistant (NDE1^SR^) and NDE1 deletion mutant lacking the DIC-binding region (NDE1ΔBR) were generated by KOD -Plus- Mutagenesis Kit (Toyobo) using the following forward/reverse primers: NDE1^SR^, 5′- AAGCGTTAAAGAAAAATTCGAGATGCAGCACTCAGAGGGT-3′/5′-TCCAGCTCCATGCGAAGGCGGTTATTCTCT-3′; and NDEΔBR, 5′-GCGCAGACGAAAGCCATTAAAGACCAACTG-3′/5′-CATCTCGAGATCTGAGTACTTGTACAGCTC-3′ using pEGFPC3/NDE1 WT as a template according to the manufacturer’s instructions. For the experiments in primary cultured oligodendrocytes and cocultures of oligodendrocytes and DRG neurons, pmaxGFP coding copGFP (Nucleofector™ Kits for Mammalian Glial Cells; Lonza) and copGFP-tagged NDE1 BR/NDE1^SR^/NDE1ΔBR expression vectors were used. For their construction, NDE1 BR/NDE1^SR^/NDE1ΔBR were subcloned into pcDNA3.1(+) (Invitrogen). The copGFP coding sequence was amplified from the pmaxGFP vector using the forward/reverse primers 5′-ATGCCCGCCATGAAGATCGAGTGCC-3′/5′-GGCGAATGCGATCGGGGTCTTGAAG-3′, and inserted into the 5′ end of NDE1 BR/NDE1^SR^/NDE1ΔBR using in-fusion methods (Takara). Mouse DIC cDNA was amplified by PCR using the forward/reverse primers 5′-ATGTCTGACAAGAGCGACCTAAAGGC-3′/5′-CAGACTCATCTCATGTCTAGGCAGC -3′, and then subcloned into the pGEM-T vector. DIC cDNA coding full protein was tagged with the HA sequence at the 5′ end and cloned into the pcDNA3.1(+) vector.

### Cell culture and transfection

HEK293T cells were cultured in Dulbecco’s modified Eagle’s medium (DMEM) containing 10% fetal bovine serum (FBS). FBD-102b cells (a kind gift of Dr. Y. Tomooka, Professor of Tokyo University of Science) were maintained in a 1:1 mixture of DMEM and F-12 medium containing 10% FBS. To differentiate FBD-102b cells, cells were cultured in a 1:1 mixture of DMEM and F-12 medium containing 1% N2 (Thermo Fisher Scientific). To transfect the cells mentioned above, Lipofectamine 2000 (Invitrogen) was used according to the manufacturer’s instructions.

### Primary cultures of rat oligodendrocytes and transfection

Primary rat oligodendrocyte cultures were performed as described previously^15^. Mix glial lineage cells were mechanically removed by shaking culture flasks at 200 rpm for 20 h, and OPCs were collected by plating the cells on a non-coated dish to remove astrocytes and microglia. For the transfection, nucleofection was performed using the Basic Nucleofector™ Kits for Primary Mammalian Glial Cells (Lonza) according to the manufacturer’s protocol. In brief, 3 × 10^5^ OPCs were resuspended in 100 μL Nucleofector™ solution per sample, and transfected with 0.5 µg expression vector and/or 1 µM siRNA using Nucleofector™ program A-33. Following transfection, cells were suspended in a 1:1 mixture of DMEM and F-12 medium containing 10% FBS and plated on four-well plates containing coverslips coated with PLL. Four hours after transfection, transfected OPCs were differentiated by replacing all of the media with Neurobasal differentiation medium (Invitrogen) supplemented with B27 (Invitrogen), 2 mM l-glutamine (Sigma), 5 ng/mL insulin (Sigma), 5 ng/mL NT3 (Pepro Tech Inc.), 15 nM triiodothyronine (Sigma), 10 ng/mL ciliary neurotrophic factor (CNTF) (Pepro Tech Inc.), and 5 µg/mL *N*-acetyl-l-cysteine (Sigma).

### Co-cultures of oligodendrocytes and DRG neurons

Primary rat oligodendrocytes and mouse DRG neuron co-cultures were prepared as described previously with some modification^49^. In brief, embryonic DRG neurons were obtained from E13.5 mice and dissociated with 0.25% trypsin for 45 min at 37 °C. The dissociated cells were seeded in four-well plates containing coverslips coated with PLL and growth factor-reduced matrigel (BD) at a density of 4 × 10^4^ cells/well in Neurobasal medium (Life Technologies) supplemented with 2% B27, 2 mM l-glutamine, 4 g/L d-glucose, and 50 ng/mL nerve growth factor (NGF) (Millipore). The DRG neurons were then cultured for 28 days, and half of the medium was replaced with supplemented Neurobasal medium every other day. To remove dividing cells, such as fibroblasts, the medium was replaced with supplemented Neurobasal medium containing 10 µM fluorodeoxyuridine (Sigma) on days 2 and 6 after seeding. After 28 days, transfected OPCs were added to DRG neurons. Four hours after seeding, co-cultures were differentiated by replacing all the media with co-culture medium (1:1 DMEM/F-12 and Neurobasal media supplemented with 1% N2, 2% B27, 2 mM l-glutamine, 10 ng/ml biotin, and 15 nM triiodothyronine).

### Quantitative real-time polymerase chain reaction

Quantitative reverse transcription polymerase chain reaction (qRT-PCR) was performed as described previously^14^. Total RNA was reverse-transcribed using the High-Capacity cDNA Reverse Transcription Kit (Applied Biosystems) and analyzed by real-time PCR to evaluate the expression of NDE1 and GAPDH. For the rat genes, the following sets of forward/reverse primers were used: NDE1, 5′- CAGCATTCAGAGGGTTACCG-3′/5′-TCCAGGGACATGATTGTGGC-3′; and GAPDH, 5′-GCCTTCTCTTGTGACAAAGTGG-3′/5′-ATTCTCAGCCTTGACTGTGCC-3′. For the mouse genes, the following sets of forward/reverse primers were used: NDE1, 5′- CAGCACTCAGAGGGTTACCG-3′/5′-TCCAGGGACATGATTGTGGC-3′; and GAPDH, 5′-GTGTTCCTACCCCCAATGTG-3′/5′-AGGAGACAACCTGGTCCTCA-3′. GAPDH was used as an internal normalization control. Real-time PCR was set up using the THUNDERBIRD SYBR qPCR Mix (Toyobo). Specific ratio comparisons (gene of interest/GAPDH) were used to assess differences in transcript expression between groups.

### Western blot analyses

Western blot analysis was performed as described previously^15^. Immunoreactivity was detected by chemiluminescence using the ECL prime kit (GE Healthcare). Densitometric quantification was performed using ImageJ software (National Institute of Health) with GAPDH as a loading control.

### Immunoprecipitation

HEK293T cells were transfected with HA-DIC and GFP-NDE1 WT or GFP-tagged truncated forms of NDE1 individually or in combination. Cells were lysed in TNE buffer/1% NP40 in the presence of protease inhibitors (Roche). Prepared lysates were incubated with an anti-HA antibody overnight at 4 °C and then with rProtein G agarose (GE Healthcare) for 6 h at 4 °C. Agarose beads were then washed five times with TNE buffer. Immunoprecipitates by anti-HA antibody were subjected to western blot analysis using anti-GFP antibody.

### Immunofluorescence staining and Sholl analysis

Immunofluorescence staining of FBD-102b cells and primary cultured oligodendrocytes was performed as described previously^14^. Sholl analysis of primary cultured oligodendrocytes was performed as described previously^49^.

### RNA ***in situ*** hybridization

Murine NDE1 cDNA fragments used as templates for probe synthesis were amplified by PCR using the following forward/reverse primers 5′-ATGGAGGACTCGGGAAAGACCTTTG-3′/5′-GGGCGCAGTTCTCATTGGTCCTAAA-3′. NDE1 cDNA subcloned into the pGEM-T vector (Promega) was linearized by restriction enzymes (NdeI or NcoI). DIG-labeled cRNA probes for NDE1 were generated by *in vitro* transcription using the cDNA fragment as a template in the presence of DIG-labeled dUTP (Roche). Hybridization procedures were performed as described previously^15^. The probe signals of NDE1 were amplified by the TSA-biotin system (PerkinElmer) according to the manufacturer’s protocol and detected using streptavidin–alkaline phosphatase (PerkinElmer) and nitro blue tetrazolium (NBT)/5-bromo-4-chloro-3-indolyl-phosphate (BCIP).

### ***In situ*** hybridization combined with immunohistochemistry

For *in vivo* analysis, sections were processed first for ISH and then immunohistochemistry as described previously^15^. To co-label with Olig2, the probe signals of NDE1 were amplified by TSA Plus DNP system, and detected using Alexa Fluor 488-conjugated anti-DNP antibody. Anti-Olig2 antibody was detected using Alexa Fluor 568-conjugated anti-mouse IgG. To co-label with CC1 or PDGFRα, the probe signals of NDE1 were amplified using the TSA-biotin system and detected using streptavidin–alkaline phosphatase (PerkinElmer) and a 2-hydroxy-3-naphthoic acid-2′-phenylanilide phosphate Fluorescent Detection Set (Roche Diagnostics). Antibodies against CC1 and PDGFRα were detected using Alexa Fluor 488-conjugated anti-mouse or rabbit IgG.

### Cell count

For *in vitro* studies, three independent cultures were established and cells were counted in multiple fields of each culture.

### Statistical analyses

All data are expressed as mean ± standard error of the mean (SEM) with the number of experiments indicated by (n). Two-tailed Student’s t-test was used for pairwise comparison. Tukey-Kramer’s or Bonferroni’s post-test following analysis of variance (ANOVA) was used for multiple comparisons. A P value of < 0.05 was considered statistically significant.

### Data availability

The datasets generated during and/or analyzed during the current study are available from the corresponding author on reasonable request.

## Electronic supplementary material

Supplemental Figure
