# Supplementary material for: NDE1 positively regulates oligodendrocyte morphological differentiation
Source: Sci Rep. 2018 May 16;8:7644. doi: 10.1038/s41598-018-25898-4 (PMC5955916; doi:10.1038/s41598-018-25898-4)
Supplement: Supplementary file 1 — Supplemental Figure [file 41598_2018_25898_MOESM1_ESM.docx]

**Title**

NDE1 positively regulates oligodendrocyte morphological differentiation

**Author**

Shoko Shimizu^1^*, Yugo Ishino^1^, Masaya Tohyama^1,2^ and Shingo Miyata^1^

**Affiliations**

^1^Division of Molecular Brain Science, Research Institute of Traditional Asian Medicine,

Kindai University, Osaka-sayama, Osaka 589-8511, Japan.

^2^Osaka Prefectural Hospital Organization, Osaka 541-8567, Japan

*Corresponding author,

Shoko Shimizu, Ph.D.

Division of Molecular Brain Science,

Research Institute of Traditional Asian Medicine,

Kindai University, Osaka, Japan

377-2, Ohno-Higashi, Osaka-Sayama,

Osaka 589-8511, JAPAN

Phone ; +81-72-366-0221 FAX ; +81-72-366-6661

[**shimizu@med.kindai.ac.jp**](mailto:shimizu@med.kindai.ac.jp)

**Supplemental Figure S1**.**
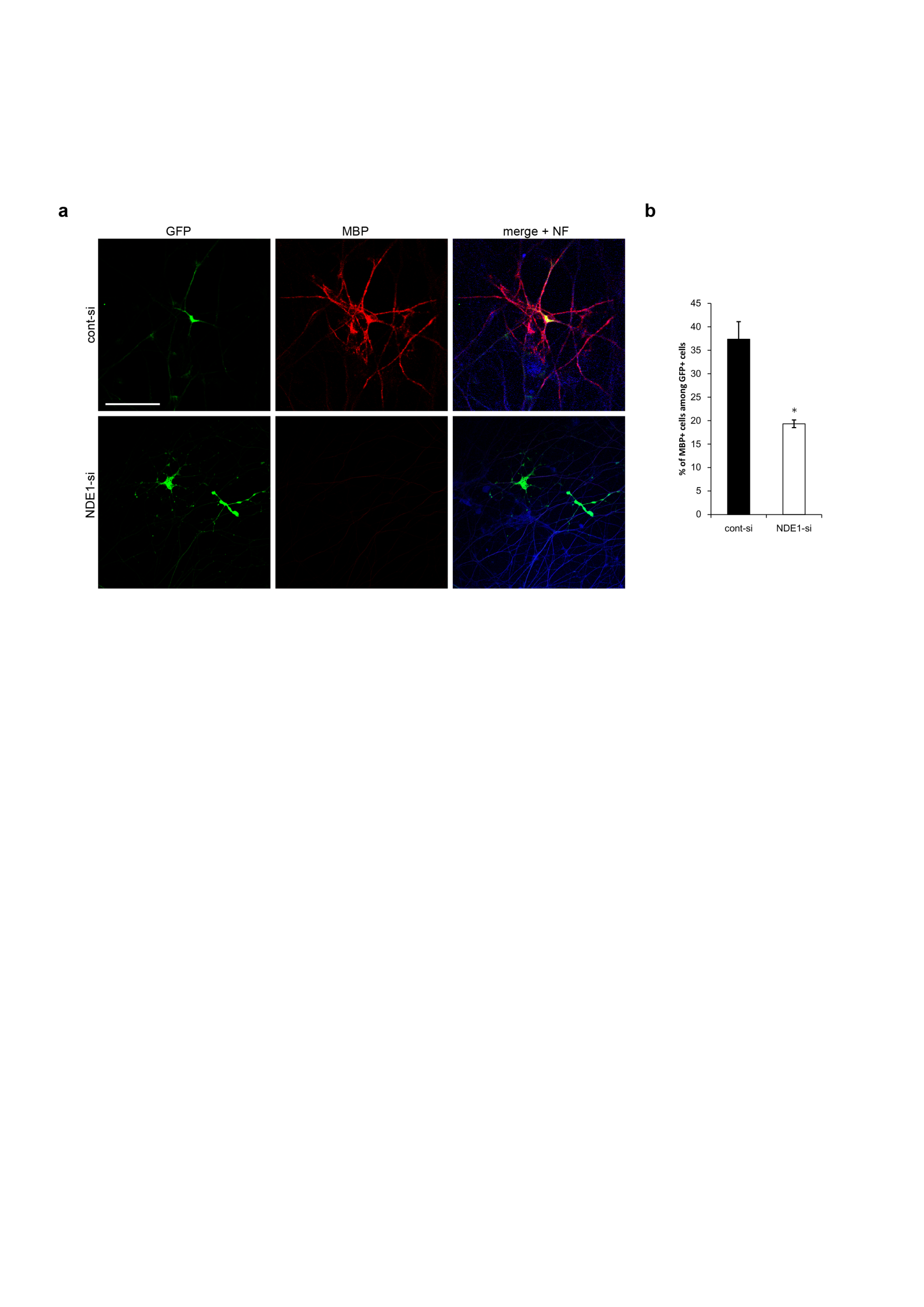
**

**Effect of NDE1 downregulation on neuron–oligodendrocyte contact formation in myelinating cocultures for 21 days.**

OPCs were transfected with NDE1 siRNA and GFP-plasmid (NDE1-si) or with control siRNA and GFP-plasmid (cont-si), added to DRG neurons, and cocultured for 21 days. (a) Immunostaining was performed using antibodies against GFP, MBP and neurofilament protein NF. (b) Quantification of MBP+/GFP+ cells from the data shown in (a). Results are the mean of more than 40 cells in total from three independent cultures. *, P < 0.01 versus cont-si (n = 3). Scale bar: 50 µm.

**Supplemental Figure S2.**

**Overexpression of DIC-binding region of NDE1 impairs process formation in FBD-102b cells.**

**
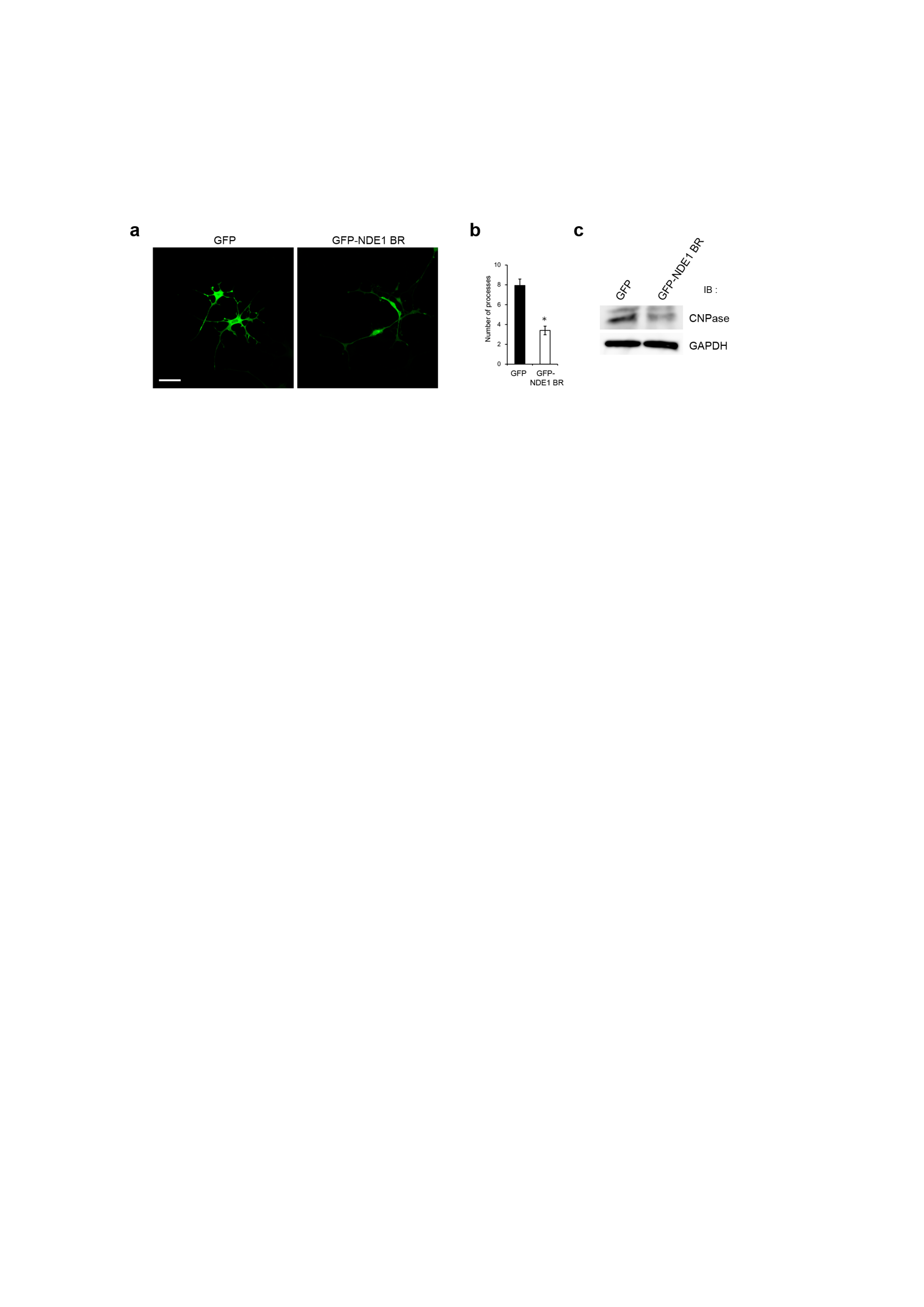
**(a) FBD-102b cells were transfected with GFP-NDE1 BR or GFP 24 h before induction of differentiation. At 48 h after induction of differentiation, cells were immunostained with antibodies against GFP. (b) The number of processes of the cells transfected with GFP or GFP-NDE1 BR is shown. Results are the mean of more than 60 cells in total from three independent cultures. *P < 0.05 versus cont-si (n = 3). (c) Lysates were prepared from cells harvested at 96 h after differentiation and subjected to western blot analysis using antibodies against CNPase and GAPDH. Scale bar: 50 µm. Full-length blots are shown in Supplementary Fig. S4k.

**Supplemental Figure S3.**

**Impaired OL process formation is not rescued with a DIC-binding region-deficient NDE1 in FBD-102b cells.**


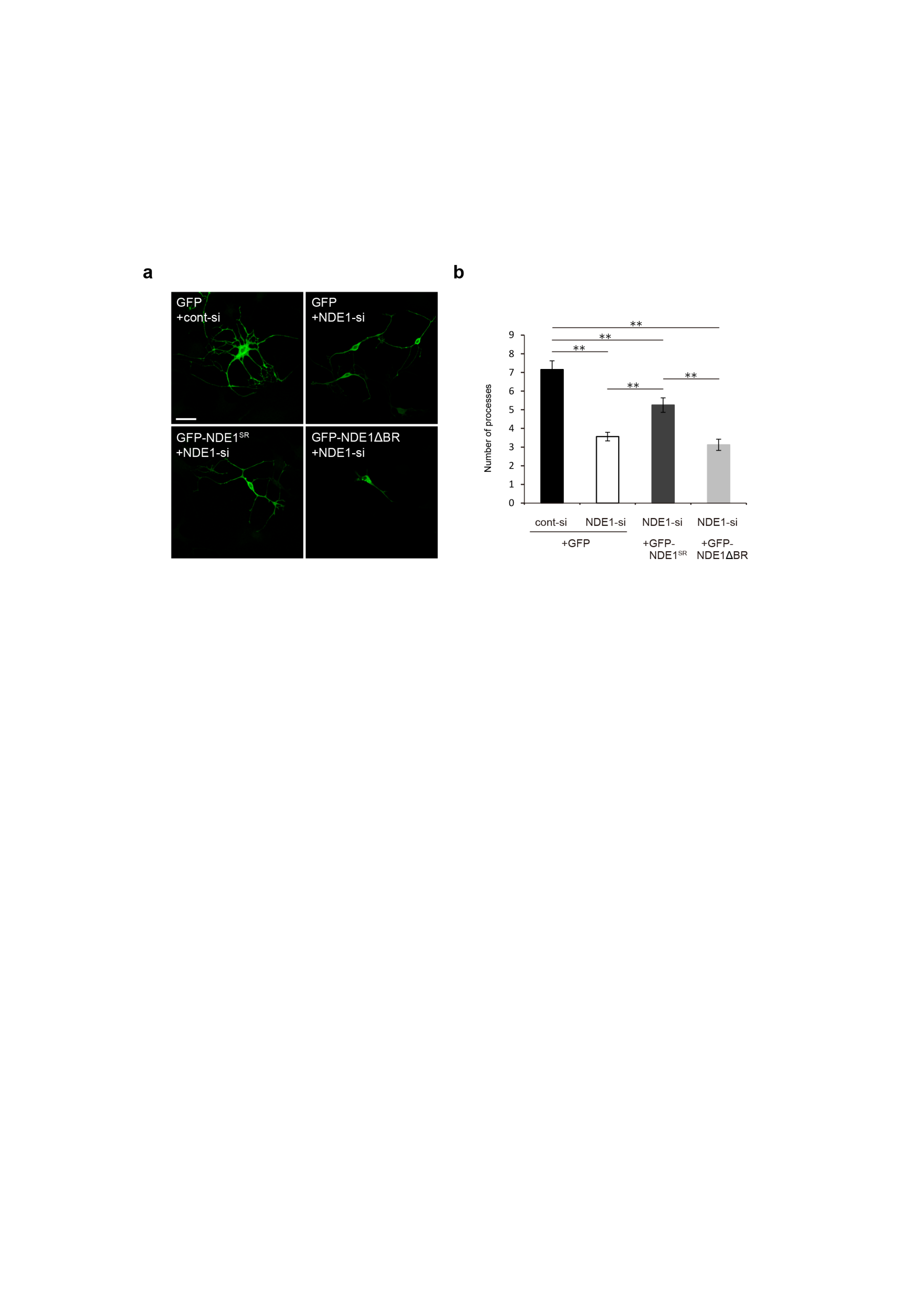
(a) FBD-102b cells were cotransfected with NDE1 siRNA (NDE1-si) or control siRNA (cont-si) and GFP, GFP-NDE1^SR^, or GFP-NDE1ΔBR in combination. At 48 h after induction of differentiation, cells were immunostained with antibodies against GFP. (b) The number of processes of the transfected cells is shown. NDE1 knockdown impaired process formation, and this phenotype was rescued by expression of GFP-NDE1^SR^, but not GFP-NDE1ΔBR (GFP+cont-si: 7.7 ± 0.29, GFP+NDE1-si: 3.9 ± 0.41, GFP-NDE1^SR^+NDE1-si: 5.1 ± 0.33, and GFP-NDE1ΔBR+NDE1-si: 3.2 ± 0.17). More than 50 cells in total from three independent cultures were counted. *, P < 0.05; **, P < 0.01 by Bonferroni’s post-test following one-way ANOVA (n = 3). Scale bar: 50 µm.


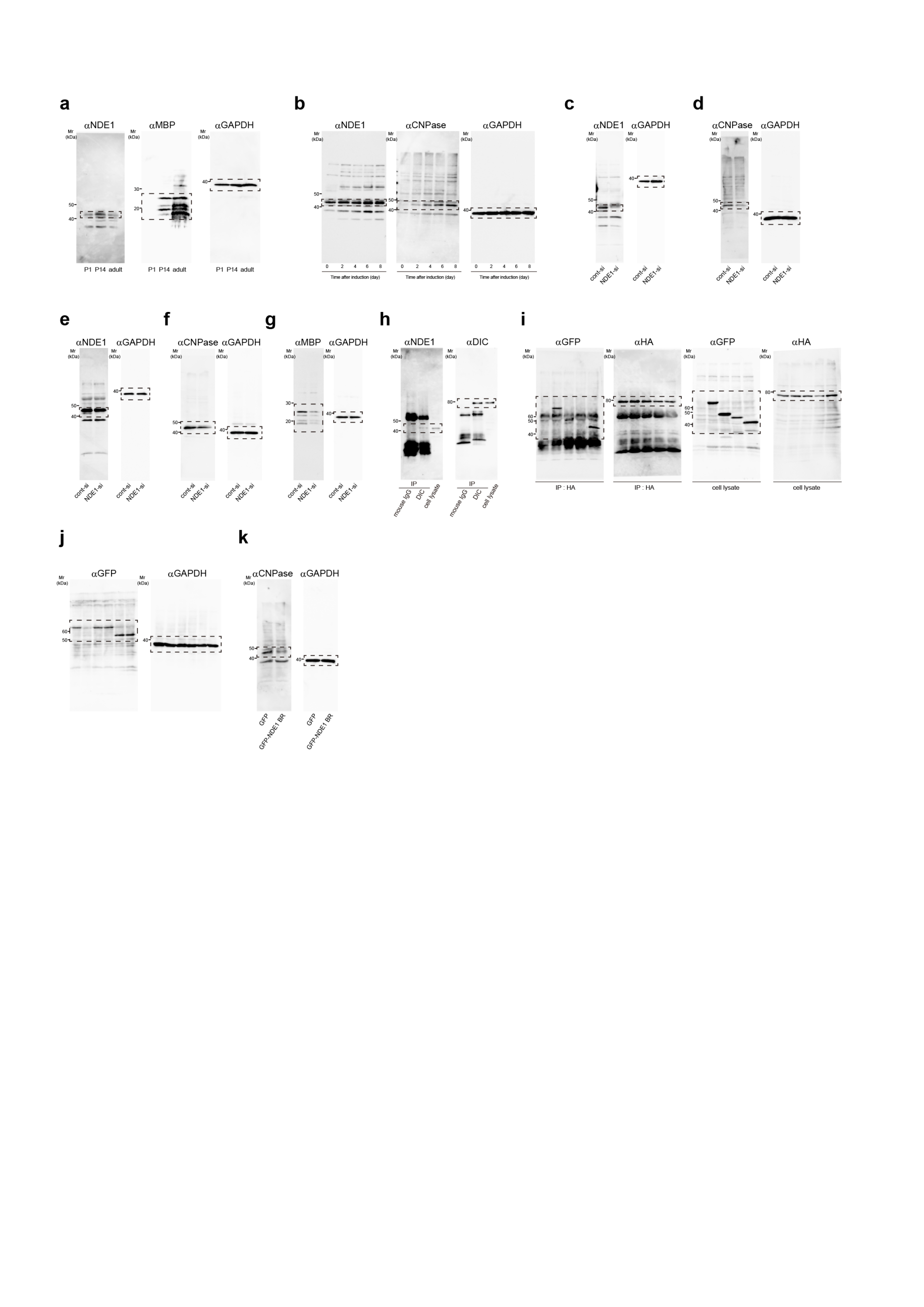


**Supplemental Figure S4.**

**Full-length images of blots presented in Figures.**

(a) Figure 1h. (b) Figure 2a. (c) Figure 2e. (d) Figure 2h. (e) Figure 3b. (f) Figure 3h. (g) Figure 3i. (h) Figure 5a. (i) Figure 5c. (j) Figure 8b. (k) Supplemental Figure S1c. Rectangles indicate the regions used in the figures.
